# Effect of Aeration Intensity on Performance of Lab-Scale Quorum-Quenching Membrane Bioreactor

**DOI:** 10.3390/membranes12030289

**Published:** 2022-03-02

**Authors:** Zia Ul Islam, Mariam Ayub, Shinho Chung, Heekyong Oh

**Affiliations:** 1 Department of Environmental Sciences, Forman Christian College (A Chartered University), Lahore 54600, Pakistan; ziaislam@fccollege.edu.pk (Z.U.I.); mishimariam7@gmail.com (M.A.); 2 School of Environmental Engineering, University of Seoul, Seoul 02504, Korea; heekyong.oh@uos.ac.kr

**Keywords:** membrane bioreactors, biofouling mitigation, quorum quenching (QQ), *Rhodococcus* sp. BH4, cell-immobilizing beads (CIBs), aeration intensity

## Abstract

Biofouling is one of the main drawbacks of membrane bioreactors (MBRs). Among the different methods, the quorum-quenching (QQ) technique is a novel method as it delays biofilm formation on the membrane surface through disruption of bacterial cell-to-cell communication and thus effectively mitigates membrane biofouling. QQ bacteria require a certain concentration of dissolved oxygen to show their best activities. Despite the importance of the amount of aeration, there have not been enough studies on aeration condition utilizing the separate determination of pure QQ effect and physical cleaning effect. This research aimed to find the optimum aeration intensity by separation of the two effects from QQ and physical cleaning. Three bead type conditions (no bead, vacant bead, and QQ beads) at three aeration intensities (1.5, 2.5, and 3.5 L/min representing low, medium, and high aeration intensity) were applied. From the results, no QQ effect and small QQ effect were observed at low and high aeration, while the greatest QQ effect (48.2% of 737 h improvement) was observed at medium aeration. The best performance was observed at high aeration with QQ beads having a 1536 h operational duration (303% improvement compared to the no bead condition); however, this excellent performance was attributed more to the physical cleaning effect than to the QQ effect.

## 1. Introduction

Biofouling is one of the main constraints associated with membrane bioreactors (MBRs) as it increases the operational costs and energy usage [1]. The quorum sensing (QS), bacterial cell-to-cell communication process is responsible for the formation of cake layers (biofilms) on membranes resulting in biofouling [2]. It is a complex process which involves the development of biofilm layers on the surfaces of membranes by producing autoinducers known as acyl homoserine lactones (AHLs) [3]. As the bacteria communicate, the concentration of AHLs increases in the MBRs and results in the deposition of cake layers on the membranes [4]. It causes a rise of transmembrane pressure (TMP) until it reaches a point where the filtration slows down or even stops in some cases [5]. This stage is called membrane biofouling [6]. At this point, the membrane modules are fouled and need to be cleaned by stronger methods or replaced with new ones.

Different methods have been used to control or inhibit biofouling, which include relaxation, standard backwash (SBW), and chemical backwash [7]. In standard backwash (also called back pulse or back flush), clean water (usually the permeate of the MBRs) is pumped in the reverse direction of the permeate through membranes. The water moving through the membranes removes the loosely attached cake layers from the surface of the membranes [8]. In chemical backwash (or chemically enhanced backwash), an oxidizing solution, such as sodium hypochlorite, is pumped in the reverse direction of the membranes with low concentrations. The chemical backwash method is known to be effective in removing irreversible fouling [9]. However, these physical/chemical methods are considered not very effective as they only remove loosely attached cake layers (standard backwash), and in some case, the cake layers become resistant to chemicals (chemical backwash) and remain attached to membranes, thus promoting further biofouling [10].

Quorum quenching (QQ) is a relatively new and novel technique in mitigation of membrane biofouling [11]. It is considered to be a very efficient method as it disrupts the cell-to-cell communication of bacteria in the sludge [12], which is the main part of QS that is responsible for the quick formation of biofilms on membrane surfaces [13]. Two strains of bacteria, *Rhodococcus* sp. BH4 and *Pseudomonas* sp. Putida, are very effective in the disruption of QS, so they are recognized as useful QQ bacteria [14].

Previously, QQ enzymes were entrapped inside membranes to control membrane biofouling [15]. Due to the cost of consumable enzymes, researchers started entrapping living QQ bacteria, such as *Rhodococcus* sp. BH4, inside the lumen of microporous membranes [10]. High density packing of QQ bacteria resulted in loss of activity, which compelled researchers to make a new design called rotating microbial carrier frame (RMCF) [16]. Then, researchers began entrapping the QQ bacteria in freely moving objects, such as bead type media initially made of sodium alginate. Entrapment of bacterial cells in the media (also referred to as immobilization or encapsulation) has been used to protect specific bacteria from harsh environments before the development of the QQ technique in the MBR research field [17,18]. Bead-type media which entrap bacterial cells have been called cell-entrapping beads [13,19,20,21,22]; however in the study, they were named cell-immobilizing beads (CIB) instead of cell-entrapping beads as to not to cause confusion with the CEB acronym for chemi-cally enhanced backwash. QQ-CIBs made of sodium alginate alone were introduced first and showed great potential to mitigate biofouling due to the synergetic combination of the QQ effect and the physical-cleaning effect [13,23]. However, the sodium alginate beads were not stable in real wastewater [18,21,22,24]. Because poly vinyl alcohol (PVA) beads were known to have high durability and good mechanical and chemical strength [25], QQ bacteria were immobilized in PVA-alginate beads for longer use of the CIBs with real wastewater in MBRs [24]. Recently, as a further improvement of QQ media, QQ hollow cylinders [26] and QQ sheets [27] were also introduced to entrap QQ bacteria. These methods were found to be very effective in controlling membrane biofouling due to the greater surface area and greater possibility of washing foulants from the surface of membranes. However, fabrication of QQ hollow cylinders and QQ sheets are more difficult than that of QQ-CIBs. Thus, utilizing QQ-CIBs made of PVA-alginate is still useful to study the QQ effect for biofouling mitigation.

The efficiency of QQ-MBRs is dependent on the provided conditions, e.g., type and shape of encapsulation media, strain of QQ bacteria, concentration of QQ cells, aeration intensity, and so on [28]. Many important conditions need to be maintained to observe the maximum effect of the QQ phenomenon. Among these conditions, air-scouring (aeration) intensity given to the sludge of MBRs is of paramount importance [19]. This is because it provides oxygen to the bacteria in the sludge and moves foulants away from the membranes by shear force in the mixed liquor, thus increasing the lifespans of the membranes [20]. It also helps in the cleaning of membranes by the physical collision of CIBs with cake layers on the membrane surface [29]. There is a synergistic combination of QQ and physical cleaning under different cleaning conditions and aeration intensities with respect to membrane fouling and energy saving [30]. In Weerasekara’s research, air back pulsing was used as an anti-biofouling technique, which also increased the overall energy consumption [31]. How much energy is saved by using QQ-MBRs at low and higher aeration intensities was reported; however, it was not reported at which aeration intensity the maximum QQ effect is observed. QQ bacteria are aerobic in nature and require a certain concentration of dissolved oxygen to show their best activities [32]. Moreover, almost 50% of the energy usage in MBRs is associated with the aeration given to the MBRs [33]. Therefore, it is very important to quantify the specific amount of air scouring to improve the QQ effect and physical-cleaning effect in the mitigation of biofouling as well as to save on the operational cost of the MBRs. In this regard, this research aimed to find the optimum aeration intensity for the best MBR performance by determination of pure QQ effect and physical cleaning effect of QQ-CIBs separately.

## 2. Materials and Methods

### 2.1. Preparation of CIBs

CIBs were prepared according to protocols suggested by Takei [17] and Van Pham and Bach [25] with some modifications by Islam and co-workers [21]. Briefly, 8% PVA and 1% sodium alginate were mixed in a dry oven for 4 h and cooled to room temperature. The QQ bacteria, *Rhodococcus* sp. BH4 (Accession no. CP014941, Gram positive), grown on LB agar medium was harvested by centrifuge and added to the PVA-alginate mixture to have a cell concentration of 5 mg in 1 mL PVA-alginate mixture. The mixture was dropped into 4% calcium chloride and 7% boric acid solution in the form of droplets and were kept in it for 30 min. The obtained beads were then dipped in 0.5 M sodium sulphate solution for 2 h and then stored in deionized water at 4 °C. The average size (diameter) of the beads was 3.4 mm. After the freeze-drying procedure, the immobilized bacteria in the beads were coated with gold particles by a sputter coater to enhance image quality by providing conductivity. Then, they were observed in different magnification by using NOVA Nano Scanning Electron Microscope 450 to confirm the successful immobilization of QQ bacteria inside the CIB. Vacant beads were also made in the same procedure except for the cell immobilization.

### 2.2. Survival Test of Rhodococcus sp. BH4 after Completion of MBR Operation

Once the QQ-MBR operation was completed, 10 CIBs were taken out from the sludge and streaked in LB agar media to check their survival. They were washed with tap water and dipped in deionized water. Then, they were put in sterile water and punctured with an autoclaved micropipette tip. This was done for any bacterial cells inside beads to be suspended in the sterile water. In the next step, 100 µL suspension was taken and spread in a freshly prepared LB agar media by a spreader. The plates were then incubated at 30 °C for 48 h. Distinct colonies were then again streaked on separate agar plates and incubated at 30 °C for 48 h to make a pure culture. The 16S rRNA sequencing was then conducted to check if *Rhodococcus* sp. BH4 has survived in the CIBs during the MBR operation.

### 2.3. MBR Design and Basic Operational Conditions

**Figure 1:**
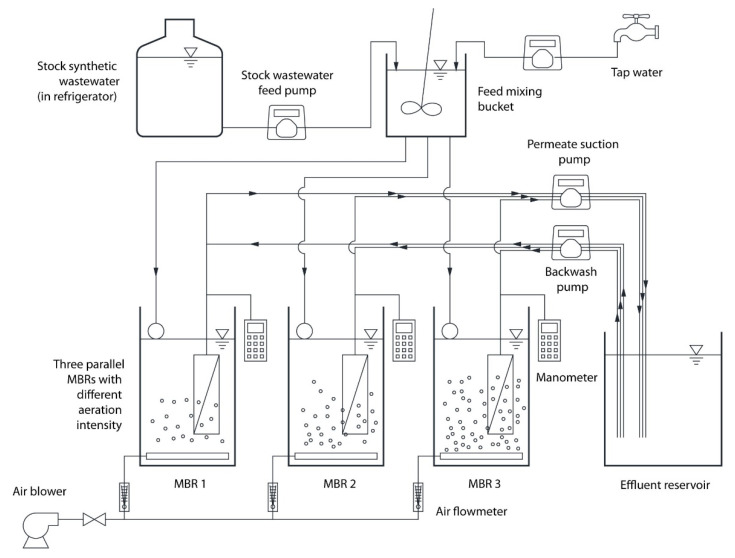
Schematic diagram of lab-scale MBR.

Lab-scale MBRs were installed in the Environmental Sciences Department of Forman Christian College (A Chartered University), Lahore, Pakistan. The design of this setup is shown in Figure 1. The setup consists of a concentrated feed bucket which is placed in a refrigerator at 4 °C. The concentrated feed is transferred to a stirring bucket where it is diluted with tap water to have a similar composition to typical domestic wastewater used in other researches [22,31], as shown in Table S1. The reactors were made of acrylic with the dimensions of 8 cm × 15 cm × 40 cm (L × W × H) to have 4 L of effective mixed liquor volume with 35 cm liquid depth. Peristaltic pumps were used for the suction of the permeate from the membrane module and for pumping the permeate backward for standard backwash. Air-scouring systems, U-shape membrane modules with hollow fiber membranes (Figure S1), and an automation system with the help of solenoid valves and a timer were set up. Effluent of MBR was stored for standard backwash and water quality tests. TMP data of each membrane module was monitored with a data-logging monometer and then transferred to a PC every week for further analysis. Hydraulic retention time (HRT), solid retention time (SRT), mixed liquor suspended solid (MLSS), and flux were selected from previous researches [31,34] on this plant. MLSS was maintained at 8000 mg/L by withdrawing excess sludge through daily checking of the MLSS concentration, and the average mixed liquor volatile suspended solid (MLVSS) was 7080 mg/L. Operational flux was set as 27 L/m^2^/h (LMH) to run the MBR on constant-flux mode. Standard backwash was applied as a basic anti-biofouling method for all the operations. All the common conditions used in this research are shown in Table 1.

### 2.4. Analysis of Water Quality Parameters

Water quality of effluent produced by MBR under different operational conditions was examined according to *Standard Methods for the Examination of Water and Wastewater,* 22nd edition [35] to check treatment efficiency. Ammonia was examined once a week, COD examined twice a week, and MLSS and MLVSS were examined daily.

### 2.5. Different Research Conditions of MBR

The research conditions of MBR were designed to observe two research variables, i.e., bead type and aeration intensity. The operational setup had three bead types with the three aeration intensities: (a) no bead condition as control (A1~A3), (b) vacant bead condition (B1~B3), and (c) QQ-CIB condition (C1~C3). To observe the anti-biofouling effect of quorum-quenching bacteria entrapped in the polymer beads, the QQ-CIB condition as well as the no bead condition as a control group were used. In the QQ-CIB condition, *Rhodococcus* sp. BH4 entrapped in PVA beads were introduced in MBRs with a 1% filling ratio of working volume of reactor. To observe the pure QQ effect that is separated from the physical cleaning effect of QQ-CIBs, comparative operations (vacant bead condition) were added. To observe the effect of aeration intensity on MBR performance, three different air flow rates were selected to be low, medium, and high, i.e., 1.5 L/min, 2.5 L/min, and 3.5 L/min with reference to previous research [22,34]. These air flow rates are equivalent to 3.1, 5.2, and 7.3 m^3^/m^2^/h as specific aeration demand (SAD_m_, air flow rate per unit surface area of membrane) and estimated to provide the velocity gradient G value of 92, 119, and 140/s, respectively, using the formulas of Mueler et al. [36] and Psoch and Schiewer [37]. The performance of MBRs was evaluated by the length of operational duration that is the time spent until the membrane module gets fouled (when TMP reaches 33 kPa, according to the maximum permissible pressure from manufacture’s guide) and the total accumulated effluent production volume. The combination of the two research variables with specific conditions are summarized in Table 2.

## 3. Results and Discussion

### 3.1. Successful Immobilization of Rhodococcus sp. BH4 in CIBs

**Figure 2:**
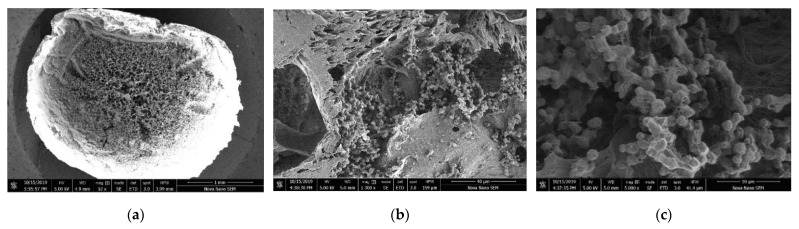
SEM images of QQ-CIBs taken in this research; (**a**) cross-section of the bead, 52×; (**b**) immobilized QQ bacteria, 1300×; (**c**) 5000×.

The SEM images taken from the CIBs are shown in the Figure 2. At the magnification of 5000×, rod-shaped *Rhodococcus* sp. BH4 were clearly seen on the inner structure of CIBs. Through the SEM examination, successful immobilization in the initial stage of *Rhodococcus* sp. BH4 in CIBs was confirmed. However, the observation of the existence of rod-shaped cells does not guarantee their aliveness or activity. These need to be confirmed by another method, i.e., through performance improvement of MBR and the survival test in this study.

### 3.2. Survival of Rhodococcus sp. BH4 upon Completion of MBR Operation 

Agar plates made from the suspension of punctured CIBs showed five different types of bacterial colonies (Figure S2). Each distinct colony was then streaked again on a fresh separate plate for the isolation of the separate strain. After 48 h incubation, bacterial colonies were grown in each plate (Figure S3). The pure cultured agar plates were sent to Macrogentech (South Korea) for 16S rRNA sequencing. The results obtained were then run on the basic local alignment search tool (BLAST) to check their similarity with *Rhodococcus* sp. BH4. It was found that bacteria grown on two out of five agar plates showed 100% nucleotide similarity with *Rhodococcus* sp. BH4 for both the 785F primer and the 907R primer (Figures S4 and S5). The other strains in the beads that had come inside the beads and grown after the beginning of the MBR operation were *Sphingomonas* sp. and *Sphingopyxis* sp. This shows that the QQ bacteria, *Rhodococcus* sp. BH4, entrapped in PVA beads were still alive until the completion of the operation (more than two months) without disappearing. The effectiveness of the CIB-making method to protect them from the harsh environment in a long MBR operation was proven by this survival test. Although this survival test is not a quantitative measure for checking how many cells are alive or dead but a qualitative one for confirming the existence of living cells, it would be enough to show that the QQ bacteria were protected and kept alive in the CIBs.

### 3.3. Analysis of Water Quality Parameters

Analysis of effluent produced under different operational conditions showed that the treatment efficiency shown by COD and ammonia nitrification was fairly good without meaningful differences between different operational conditions applied in this research. The average removal efficiencies for all the operations were 90.5~94.5% for COD (Figure S6) and 96.2~99.6% for ammonia (Figure S7), as shown in Table 3. According to the result of the one-way ANOVA to check for the existence of meaningful differences between the average removals of different operations, the minor differences turned out to be statistically insignificant as the *p*-values for both COD and ammonia are much greater than 0.05. Thus, it can be said that the introduction of vacant beads to MBRs for physical cleaning and QQ-CIBs for disruption of cell-to-cell communication of bacteria both did not provide any negative nor positive effect on the treatment ability of bacteria in terms of COD and ammonia removal. Furthermore, it was observed that low, medium and high aeration intensities applied in this research did not increase nor decrease the removal efficiencies of COD and ammonia. This result was considered desirable because the introduction of the QQ technique in MBRs was to improve the performance of MBRs in terms of operational duration by mitigation of biofouling rather than to improve wastewater treatment efficiency. Thus, it is now reasonable to compare the operational duration of MBRs apart from wastewater treatment quality.

### 3.4. Comparisons of Different Operations of MBRs

**Figure 3:**
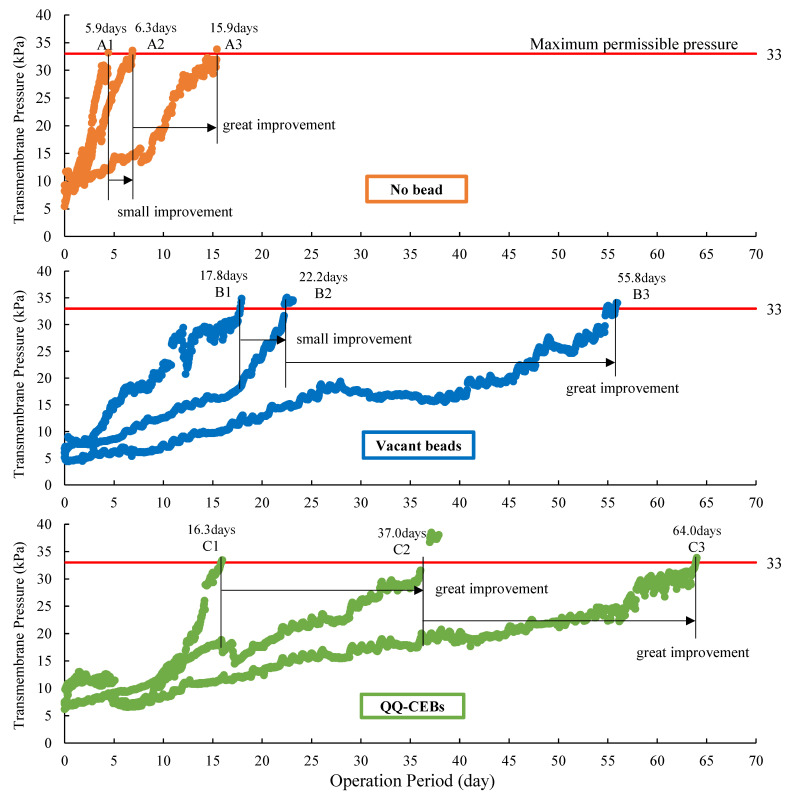
TMP profiles of each operation.

The performance of MBRs was evaluated by operational duration (time spent until the membrane module got fouled) through monitoring transmembrane pressure. As expected, it was observed that the no bead condition got fouled early, the vacant bead condition got fouled late, and the QQ-CIB condition got fouled later, as shown in the TMP profiles of each operation (Figure 3). The A1 operation lasted 5.9 days with the production of 80 L of effluent. The A2 operation lasted 6.3 days with 86.7 L of effluent production. The A3 operation lasted 15.9 days with 216 L of effluent production.

In the A1 and A2 operations, the TMP increased rapidly indicating that standard backwash alone at low and medium aeration intensities is not enough to mitigate membrane biofouling. Therefore, it needs to be coupled with other anti-biofouling techniques for longer operations. On the other hand, the A3 operation showed a relatively longer operational duration, showing that higher aeration moves foulants away from the membrane surface thus mitigating biofouling.

The operational durations of B1, B2, and B3 were 17.8 days, 22.2 days, and 55.8 days, respectively. The effluent productions (total accumulated production volume) were 242.2 L, 302 L, and 759.5 L, respectively. In the vacant bead conditions, the TMP increased more slowly than those of the no bead conditions. The physical-cleaning effect of vacant beads showed an obvious longer operational duration as well as a greater effluent production. Similar to the result of the no bead conditions, the higher aeration intensity brought greater improvement while the medium aeration did not.

The TMP profile also showed that the C1 operation took 16.3 days to get fouled, C2 took 37.0 days, and C3 took 64.0 days. Based on the slow increase of TMP due to the greatest biofouling mitigation, the longest operation of C3 showed the greatest productivity of 870 L and followed C2 (503 L) and then C1 (221.8 L). While the operational duration of C1 was slightly shorter than that of B1, TMPs of C2 and C3 increased very slowly, showing 20.7 days of improvement and 47.7 days of improvement when compared to that of C1. One unique trend of the QQ-CIB conditions is that the medium aeration intensity brought a greater positive effect (C1→C2), while it did not bring that much positive effect in the cases of the no bead conditions (A1→A2) and the vacant bead conditions (B1→B2). This means that the medium strength aeration effectively promoted the activity of QQ bacteria from 2.5 L/min and thus increased the operational duration greatly.

### 3.5. Effect of Bead Type on MBR Performance

**Figure 4:**
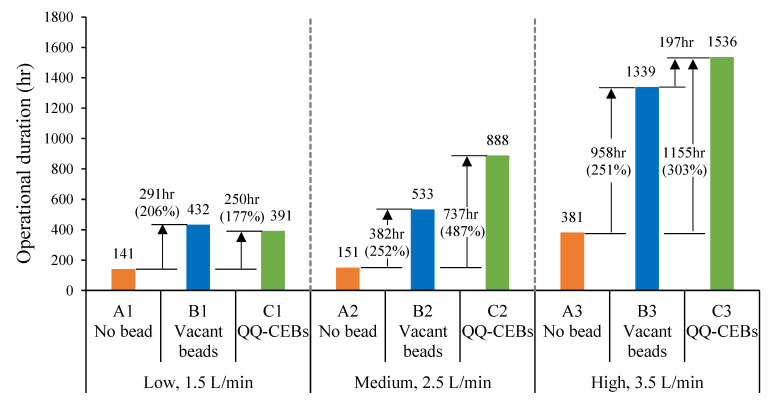
Effect of bead type on operational duration.

To check the effect from bead type, A series, B series, and C series operations at the same aeration intensity were compared (A1-B1-C1, A2-B2-C2, A3-B3-C3), as shown in Figure 4. The introduction of beads in the MBRs increased the operational duration for both vacant beads and QQ-CIBs. At the low aeration intensity, the introduction of vacant beads (B1) increased the duration by 291 h (206% improvement) compared to the no bead condition (A1). The introduction of QQ-CIBs (C1) also increased the duration; however, it was by 250 h (177% improvement), slightly smaller than that by the vacant beads. The C1 operation was expected to show longer operational duration than the B1 operation as QQ bacteria will delay biofilm formation on membranes by disrupting the QS process. However, the C1 operation (391 h) was shorter than the B1 (432 h) operation. This means that the quorum quenching effect did not take place at the low aeration. This might be because the aeration intensity of 1.5 L/min for 4 L MBR was not enough for *Rhodococcus* sp. BH4, although the MBR maintained the dissolved oxygen level at around 1.8~2.5 mg/L. Being an aerobic bacterium, *Rhodococcus* requires an optimum level of oxygen to grow well and degrade AHLs, but it might not be met inside the polymer beads by the 1.5 L/min aeration. Thus, the improvement of the operational duration at the low aeration intensity (A1→B1 and C1) are attributed to the physical-cleaning effect only because there was no improvement by the QQ effect.

At the medium aeration intensity, the introduction of vacant beads (A2→B2) increased the duration by 382 h (252% improvement) compared to the no bead condition, which is greater than that of low aeration (A1→B1). The introduction of QQ-CIBs (A2→C2) greatly increased the duration by 737 h (487%). It means the expected QQ effect, mitigation of biofouling by disruption of signal molecules, began working from 2.5 L/min aeration intensity. These improvements at medium aeration are attributed to physical cleaning as well as the QQ effect for the mitigation of biofouling.

At the high aeration intensity, the introduction of vacant beads (A3→B3) increased the duration by 958 h (251% improvement) compared to the no bead condition. The introduction of QQ-CIBs (A3→C3) increased the duration by 1155 h (303% improvement). The improvement in the hours of the duration at the high aeration intensity were very great; however, the percentage improvements were not as great as those of medium aeration. Additionally, a large portion of improvement at high aeration is attributed to physical cleaning rather than the QQ effect because the additional improvement by QQ (197 h, B3→C3) is relatively small.

### 3.6. Effect of Aeration Intensity on MBR Performance

**Figure 5:**
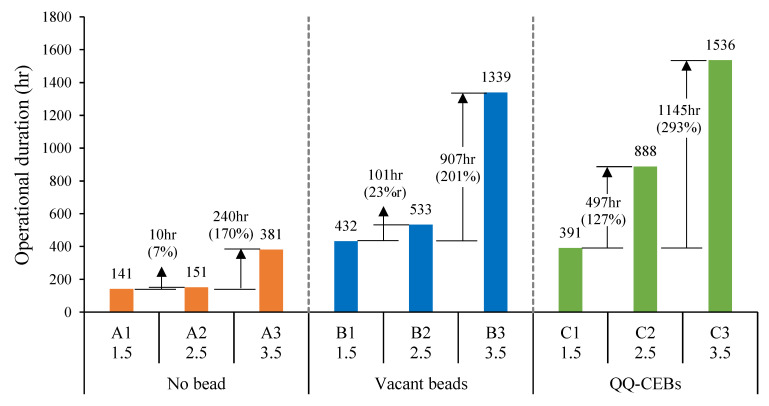
Effect of aeration intensity on operational duration.

To check the effect of aeration intensity, the different aeration intensities with the same bead type were compared (A1-A2-A3, B1-B2-B3, C1-C2-C3), as shown in Figure 5. The improvement of operational duration by increased intensity of aeration was obvious. In the absence of the QQ effect (no bead and vacant bead conditions), 1 L/min increment of aeration from 1.5 to 2.5 L/min brought minor improvements to the performances, i.e., 10 h (7%) and 101 h (23%) for the no bead (A1→A2) and vacant beads condition (B1→B2), respectively. However, the further 1 L/min increment of aeration from 2.5 to 3.5 L/min (total 2 L/min from 1.5 to 3.5 L/min) brought a great improvement, i.e., 240 h (170%) and 907 hr (210%) for the no bead (A1→A3) and vacant beads condition (B1→B3), respectively. This means 1.5 L/min of aeration is not enough to provide shear force to detach the foulant from the membrane surface effectively, and 2.5 L/min can provide some force to show a small improvement in the vacant bead condition but still not enough. 3.5 L/min of aeration as high aeration intensity is sufficient to provide moving forces and results in greater shear force, and greater movement and collision of beads with membranes. Thus, in turn, it increases the physical cleaning of membranes and delays biofouling. Therefore, when vacant beads are used for mitigation of biofouling, it is obviously efficient to use higher aeration based on the point of view of efficiency of anti-biofouling without consideration of the cost.

On the other hand, in the presence of the QQ effect (QQ-CIB conditions), a 1 L/min increment of aeration from 1.5 to 2.5 L/min (C1→C2) brought great improvement, 497 h (127%), and a further 1 L/min increment of aeration from 2.5 to 3.5 L/min (C1→C3, total 2 L/min from 1.5 to 3.5 L/min) again brought great improvement, 1145 h (293%). It is due to the promotion of the activity of QQ bacteria by aeration at 2.5 L/min, which had not shown good improvement in the A2 and B2 operations. Medium aeration (C2) showed greater improvement not only by activation of the QQ effect, but also by promotion of the physical cleaning effect. Since *Rhodococcus* sp. BH4 is an aerobic bacterium, it needs an adequate level of dissolved oxygen to grow well and degrade AHLs inside the beads. Thus, the 2.5 L/min of aeration was thought to be adequate to activate the bacteria to show a proper QQ effect in this research condition.

### 3.7. Separation of QQ Effect from Combined Effect

**Figure 6:**
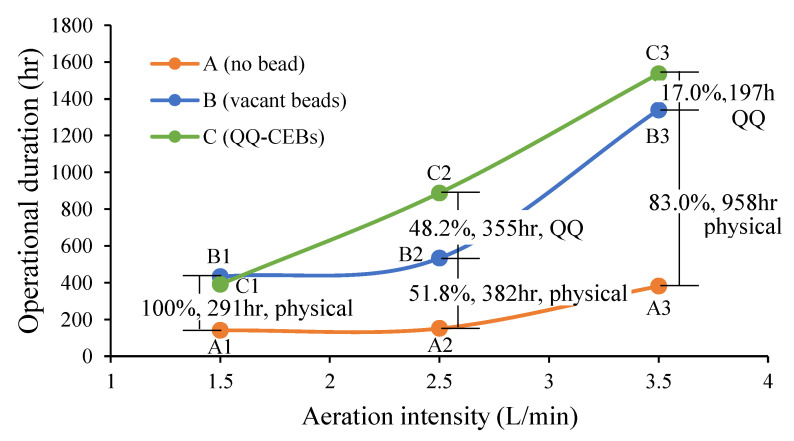
Performance improvement by pure QQ effect and physical cleaning effect and their contribution (%).

The operational durations of three bead types (no bead, vacant beads, and QQ-CIBs) along with three aeration intensities were plotted in Figure 6 to show the pure QQ effect separated from the combined effect of QQ and physical cleaning. The operational durations of no bead and vacant beads did not increase a lot until 2.5 L/min but increased a lot at 3.5 L/min to show great improvement. However, the operational duration of QQ-CIBs began increasing from 1.5 L/min linearly until 3.5 L/min. After the separation of the pure QQ effect from the combined effect, 2.5 L/min was found to have the maximum QQ effect (355 h) of 48.2% out of the total improvement of 737 h. On the other hand, 3.5 L/min had a smaller QQ effect (197 h) of 17% out of the total. Unexpectedly, the higher aeration did not show a greater QQ effect. This might be because the higher aeration tends to break large flocs in mixed liquor into small pieces and increase the concentration of soluble microbial products (SMPs). As the concentration of SMPs increases, the QQ effect is compromised and fouling of membranes increases. This result follows the findings suggested by Iorhemen and fellow researchers [38]. This result is also in accordance with a study done by Weerasekara which suggests that higher aeration removes the biofouling layer efficiently from membranes but does not allow QQ bacteria to disrupt QS signals; thus, it reduces the performance of QQ-MBRs [31]. Hence, it was concluded that the improvement in the QQ-CIB operation at the higher aeration intensity is mainly attributed to physical cleaning (83% of total improvement) rather than the QQ effect (17%), while the improvement at the medium aeration intensity is attributed to both physical cleaning and the QQ effect under the research conditions applied in this study.

In Weerasekara and his co-workers’ research [31], three aeration intensities were applied as low, medium, and high intensities as the velocity gradient (G = 51, 72 and 103/s). These G values are equivalent to 0.5, 1, and 2 L/min (0.03, 0.06 and 0.12 m^3^/h) as well as SAD_m_ 3.19, 6.38, and 12.77 m^3^/m^2^/h (calculated from the data provided). There was a good QQ effect at the low aeration but no significant QQ effect at the high aeration. The reason why the QQ effect was observed at the low aeration while there was no QQ effect at the high aeration might be due to the MLSS concentration of 6000 mg/L (lower than that of this research, 8000 mg/L), and the MBRs were not operated until the membrane got fouled. Their velocity gradients (G = 51, 72 and 103/s) are smaller than those of this research (G = 92, 119, and 140/s) while their SAD_m_ (3.19, 6.38, and 12.77 m^3^/m^2^/h) are greater than those of this research (SAD_m_ = 3.11, 5.19, and 7.27 m^3^/m^2^/h), which means that due to the small size of the reactor with a shallow depth in spite of the slightly greater amount of air, their mixing effect by air (G value) were not great. Thus, the fact that no QQ effect was observed in this research at MLSS 8000 mg/L and the fact that the QQ effect was observed at MLSS 6000 mg/L at a low aeration intensity with a similar SAD_m_ is reasonable. However, the result of the no QQ effect at the high aeration intensity in their study might be due to too high of a SAD_m,_ which might mask the QQ effect or too short of an operation, possibly causing the no observation of the possible QQ effect in the later part of the operation. In Kim and his co-workers’ research [13], the performance of MBRs with no bead, vacant beads, and QQ-CIB conditions were compared until the end of the operation and a good physical cleaning effect and QQ effect were observed. However, the aeration intensities were not reported, and the QQ-CIBs were made of sodium alginate only. In other research of Kim and his co-workers’ [18], better quality of CIBs was made by the phase inversion method to coat the sodium alginate bead with polymers to improve the durability of CIBs. The performance of MBRs with vacant beads and QQ-CIBs were compared until the end of operation; however, the aeration intensity was not reported. In Lee and his co-workers research [24], vacant beads and QQ-CIBs made of PVA-alginate were compared with real wastewater in a pilot-scale plant. A good QQ effect separated from the combined effect was observed; however, the aeration intensity was only one, i.e., 0.3 m^3^/m^2^/h of SAD_m_ for air scouring. Because an additional 10 L/min of air was supplied for the provision of oxygen apart from air scouring, the total SAD_m_ would be 0.97 m^3^/m^2^/h. Still, at the low aeration, the QQ effect was observed, but it is difficult to compare the result with ours because of no change in aeration intensity as well as the different wastewater quality and plant scale. Huang and his co-workers [19] studied two aeration intensities with vacant beads and QQ-CIBs made of PVA-alginate in study conditions most like our research. However, they focused more on sludge properties rather than MBR performance, so they did not operate the MBR until the end when the membrane gets fouled. Hence, they could not find a significant difference between the vacant bead and QQ-CIB conditions but found some only between the low and high aeration.

Thus, in the aforementioned studies, the QQ effect was not determined separately from the combined effect utilizing QQ-CIBs made of PVA-alginate at different aeration intensities. If there was no observation of the effect of vacant beads (blue line in Figure 6), the effect of QQ-CIBs (green line in Figure 6) must be thought to have the greatest improvement at the higher aeration intensity without consideration of physical cleaning included in the combined effect. However, here, due to the exclusion of the physical effect from the combined effect, it was found that the pure QQ effect at high aeration was smaller than medium aeration. Hence, the maximum QQ effect has taken place at 2.5 L/min to show maximum improvement of 355 h (48.2%) out of a total of 737 h of improvement. It suggests that the maximum QQ effect may happen at a certain optimum level of aeration, which may not coincide with the highest aeration, while the maximum physical cleaning effect by polymer beads happens with the highest aeration.

The result of the current study suggests that a decision should be made among the choices whether physical cleaning should be maximized by increasing aeration with a greater energy cost or the QQ effect should be maximized by adding the effort of making QQ-CIBs with bacterial cultures and the encapsulation cost. When it is assumed that a longer operational duration leads to a less extreme condition to the membrane (i.e., experience of fouling and recovery cleaning) and thus requires less replacement of the membrane when it is not possible to recover their performance at all, the long term relative cost from membrane replacement of QQ-CIBs (C3) will be inversely proportional to the relative improvement of operational duration, i.e., around 1/3 of the no bead operation (C1) [34]. However, this relative cost does not include the energy cost, the bacterial culture, or the fabrication of the QQ-CIBs. Therefore, an extensive cost-benefit analysis on the improvement of MBR performance using QQ-media should be conducted for wider application in the field.

## 4. Conclusions

The QQ-CIB method used in this research was found to be very efficient. The results of the SEM examination and 16S rRNA sequencing showed a successful initial immobilization and successful survival at the end of operation. The immobilized bacteria, *Rhodococcus* sp. BH4, was alive in the beads until the end of the two months operation.

The introduction of QQ-CIBs showed great potential in mitigation of biofouling by disrupting the QS process of bacteria in the MBR as well as providing the physical cleaning effect by collision of the beads against the biofilm on the membrane surface. The longest operational duration was observed in the MBR with 1% of QQ-CIBs combined with the periodic standard backwash at 8000 mg/L MLSS at the high aeration intensity (3.5 L/min) to have 1536 h of operational duration with the greatest effluent production (870 L). QQ-CIBs improved the operational duration by 1155 h (303% improvement) when compared to the no bead operation (381 h). However, this excellent performance is attributed more to the physical cleaning effect (83% contribution on a total of 1155 h of improvement) than the QQ effect (17% contribution).

After separation of the pure QQ effect from the combined effect of disruption of QS and physical cleaning of the beads, it was found that the activity of QQ bacteria is highly dependent on the specific aeration intensity. No QQ effect was observed at the low aeration intensity (1.5 L/min) and a small QQ effect was observed at the high aeration intensity (3.5 L/min). At the medium aeration intensity (2.5 L/min), the QQ effect was the greatest, showing a 335 h extension of the operational duration (48.2% of the total 737 h of improvement), which was attributed to the activity of the QQ bacteria alone. However, the physical cleaning effect of the beads was affected simply by the aeration intensity. Therefore, it is concluded that the optimization of the aeration intensity rather than a simple increment is needed in order to maximize the QQ effect (optimum level in the conditions of this study: air flow rate = 2.5 L/min, SAD_m_ = 5.11 m^3^/m^2^/h, G = 92/s), whereas a simple increment of aeration is enough in order to maximize the physical cleaning effect (air flow rate = 3.5 L/min, SAD_m_ = 5.19 m^3^/m^2^/h, G = 140/s).

## Figures and Tables

**Table 1:** Basic operational conditions of the lab-scale MBR.

Parameters	Description or Values	Parameters	Description or Values
Type of MBR	Single stage, submerged	Manufacturer	PHILOS Korea
Working volume	4 L/reactor	Membrane material	Hydrophilic PVDF
Permeate flow rate	13 mL/min	Pore size	0.1 µm
Backwash flow rate	26 mL/min	Module design	U-shape
SBW frequency	1 min after every 10 min	Membrane dimension	φ 2.3 mm, length 50 cm, 8 fibers
HRT	5.1 h	Effective surface area	289 cm^2^/module
SRT	17~20 days	No. of module	1 module/reactor
MLSS	8000 mg/L	Flux	27 L/m^2^/h
MLVSS	7080 mg/L	Influent COD	205~250 mg/L
Food/Microorganism	0.12~0.15 gCOD/gVSS·day	Influent Ammonia-N	26~33 mg/L

**Table 2:** Different research conditions of MBRs.

Operation Names	Research Variable1: Bead Type	Research Variable2: Aeration Intensity (L/min)	SAD_m_ (m^3^/m^2^/h)	Velocity Gradient G (/s) ^a^	Beads Size φ (mm)	Filling Ratio of Beads in Reactor (%)
A1	No bead	1.5	3.1	92	-	-
A2	No bead	2.5	5.2	119	-	-
A3	No bead	3.5	7.3	140	-	-
B1	Vacant beads	1.5	3.1	92	3.4	1%
B2	Vacant beads	2.5	5.2	119	3.4	1%
B3	Vacant beads	3.5	7.3	140	3.4	1%
C1	QQ-CIBs	1.5	3.1	92	3.4	1%
C2	QQ-CIBs	2.5	5.2	119	3.4	1%
C3	QQ-CIBs	3.5	7.3	140	3.4	1%

^a^ G = $\sqrt{(Q_{a}\times P_{1}\times \ln\left(\frac{P_{1}}{P_{2}}\right))/\left(3.61\times V\times \mu \right)}$ where, *Q_a_* = air flowrate (m^3^/h), *P*_1_ = absolute pressure at the mixed liquor surface, *P*_2_ = absolute pressure at the point of air injection (kPa), *V* = mixed liquor volume (m^3^), and *μ* = dynamic viscosity (Pa·s) [36,37].

**Table 3:** Removal efficiencies of COD, ammonia, and one-way ANOVA result.

Operation Name	COD			Ammonia (Nitrification)		
	Average Removal (%)	Standard Deviation (%)	n	Average Removal (%)	Standard Deviation (%)	n
A1	92.2	7.6	5	96.5	5.6	4
A2	91.9	5.8	5	99.5	0.1	4
A3	93.9	4.2	5	98.9	0.9	4
B1	92.5	7.1	9	95.5	5.0	5
B2	94.5	4.9	9	96.6	5.1	5
B3	94.6	8.5	5	96.2	5.8	4
C1	90.5	1.1	4	97.1	4.0	3
C2	93.2	10.7	10	99.6	0.5	8
C3	92.9	7.7	18	99.3	0.7	13
One-way ANOVA	*p*-value = 0.992 > 0.05			*p*-value = 0.237 > 0.05		

## Data Availability

The data presented in this study will be available on request to the corresponding authors.

## Supplementary Materials

The following supporting information can be downloaded at: https://www.mdpi.com/article/10.3390/membranes12030289/s1, Figure S1: U-shape membrane module used in the study; Figure S2: Different bacterial strains grown on agar plate; Figure S3: Five bacterial strains grown on separate agar plates; Figure S4: Sequencing result, plate no 2 (a) blast result of 785F primer; (b) blast result of 907R primer; Figure S5: Sequencing result, agar plate no 3; (a) blast result of 785F primer; (b) blast result of 907R primer; Figure S6: Removal efficiencies for COD; Figure S1: Different bacterial strains grown on agar plate; Figure S7: Removal efficiencies for ammonia; Table S1: Composition of Synthetic Wastewater as Influent of MBR.
