# Supplementary material for: Effect of Aeration Intensity on Performance of Lab-Scale Quorum-Quenching Membrane Bioreactor
Source: Membranes (Basel). 2022 Mar 2;12(3):289. doi: 10.3390/membranes12030289 (PMC8953932; doi:10.3390/membranes12030289)
Supplement: Supplementary file 1 [file membranes-12-00289-s001.zip › membranes-1591084-supplementary.pdf]

Supplemental Materials

for

Effect of Aeration Intensity on Performance of Lab-Scale Quorum-Quenching Membrane Bioreactor

Table S1. Composition of Synthetic Wastewater as Influent of MBR

Components	Value (mg/L)
Glucose	120
Peptone	90
Yeast extract	12
$(NH_4)_2SO_4$	96
$(KH_2)PO_4$	17
$NaHCO_3$	300
$CaCl_2 \cdot 2H_2O$	2.40
$MgSO_4 \cdot 7H_2O$	24
$MnSO_4 \cdot 5H_2O$	2.16
$FeCl_3 \cdot 6H_2O$	0.12
pH	7~8
BOD	130~190
COD	205~250
Ammonia-N	26~33

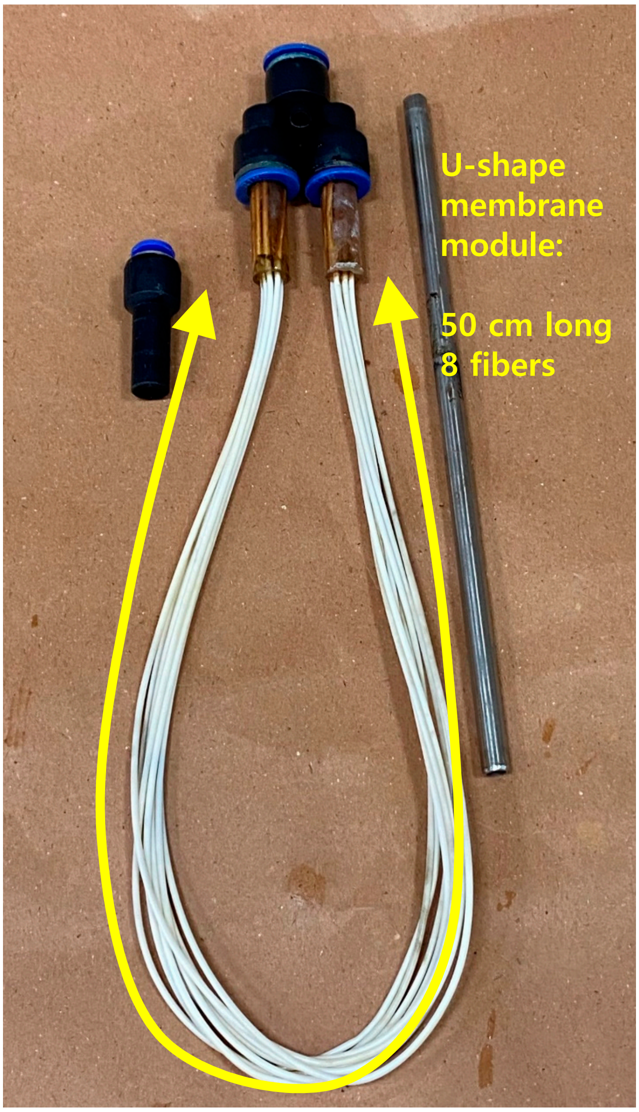

Figure S1. U-shape membrane module used in the study

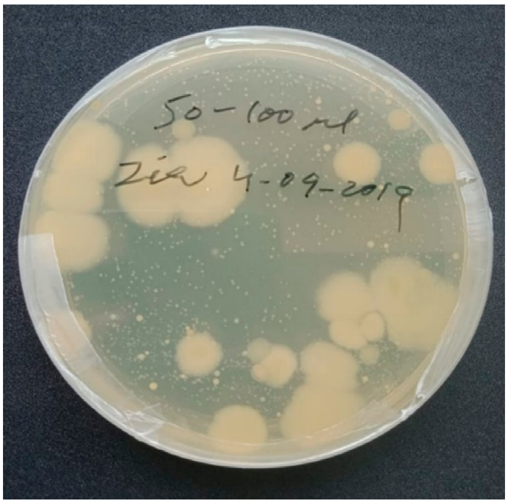

Figure S2. Different bacterial strains grown on agar plate

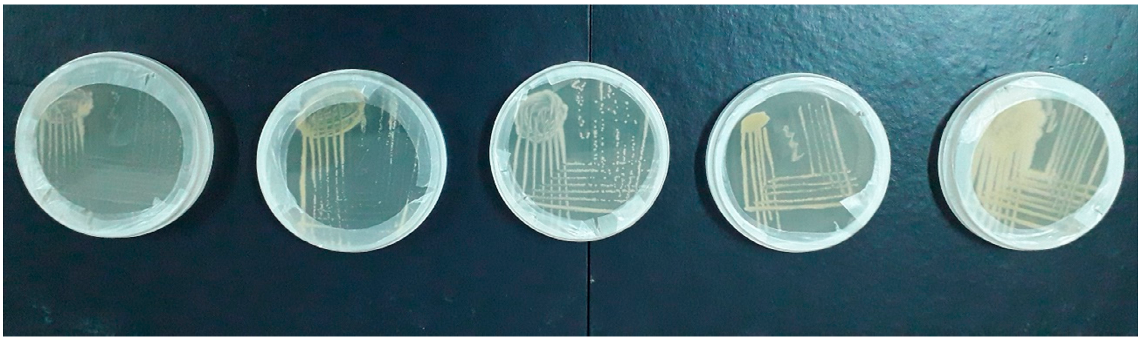

Figure S3. Five bacterial strains grown on separate agar plates

```
BLASTN 2.6.0+


Reference:
Zheng Zhang, Scott Schwartz, Lukas Wagner, and Webb Miller (2000),
"A greedy algorithm for aligning DNA sequences", J Comput Biol 2000;
7(1-2):203-14.



Database: Nucleotide collection (nt)
           43,634,103 sequences; 150,987,822,336 total letters



Query= 191015-020_H09_P2_785F 1 726

Length=726
                                                                      Score     E
Sequences producing significant alignments:                          (Bits)  Value

AY168579.1  Rhodococcus sp. HS1 16S ribosomal RNA gene, partial s...  878     0.0
AB288061.1  Rhodococcus boritolerans gene for 16S ribosomal RNA, ...  876     0.0
KY906993.1  Rhodococcus sp. strain D-46 16S ribosomal RNA gene, p...  874     0.0
JQ435727.1  Rhodococcus erythropolis strain FF9 16S ribosomal RNA...  874     0.0
MF351723.1  Rhodococcus sp. strain YL-1 16S ribosomal RNA gene, p...  872     0.0
KX156801.1  Rhodococcus sp. 17PEL clone 17PEL 16S ribosomal RNA g...  872     0.0
CP014941.1  Rhodococcus sp. BH4, complete genome                      872     0.0
LC133609.2  Rhodococcus sp. JCM 28262 gene for 16S ribosomal RNA,...  872     0.0
LC133620.2  Rhodococcus sp. JCM 28273 gene for 16S ribosomal RNA,...  872     0.0
CP017299.1  Rhodococcus sp. YL-1, complete genome                     872     0.0
```

a)

```
Query= 191015-020_J09_P2_907R 1 929

Length=929
                                                                      Score     E
Sequences producing significant alignments:                          (Bits)  Value

KF777561.1  Rhodococcus sp. M_A_09/11_5_1 16S ribosomal RNA gene,...  874     0.0
LC107442.1  Rhodococcus erythropolis gene for 16S ribosomal RNA, ...  870     0.0
KF097414.1  Uncultured bacterium clone nck237b05c1 16S ribosomal ...  870     0.0
KF923423.1  Rhodococcus qingshengii strain 13 16S ribosomal RNA g...  870     0.0
JF130075.1  Uncultured bacterium clone ncd1613b09c1 16S ribosomal...  870     0.0
JF127047.1  Uncultured bacterium clone ncd1478c09c1 16S ribosomal...  870     0.0
HQ616318.1  Uncultured Rhodococcus sp. clone T0088 16S ribosomal ...  870     0.0
DQ125599.1  Uncultured bacterium clone AKAU3602 16S ribosomal RNA...  870     0.0
KY906993.1  Rhodococcus sp. strain D-46 16S ribosomal RNA gene, p...  869     0.0
LC107441.1  Rhodococcus erythropolis gene for 16S ribosomal RNA, ...  869     0.0
LC107436.1  Rhodococcus erythropolis gene for 16S ribosomal RNA, ...  869     0.0
LC107435.1  Rhodococcus erythropolis gene for 16S ribosomal RNA, ...  869     0.0
KF777639.1  Rhodococcus sp. M_Sw_oHS_10/11_2_1(2) 16S ribosomal R...  869     0.0
EU977829.1  Rhodococcus globerulus strain 1P10AA 16S ribosomal RN...  869     0.0
LC107438.1  Rhodococcus erythropolis gene for 16S ribosomal RNA, ...  867     0.0
KF098191.1  Uncultured bacterium clone ncd1396c02c1 16S ribosomal...  867     0.0
EU368379.1  Uncultured bacterium clone 629 16S ribosomal RNA gene...  867     0.0
DQ898299.1  Rhodococcus sp. MTR-70 16S ribosomal RNA gene, partia...  867     0.0
AF487704.1  Nocardia sp. H17-1 16S ribosomal RNA gene, partial se...  867     0.0
MF351723.1  Rhodococcus sp. strain YL-1 16S ribosomal RNA gene, p...  865     0.0
MF114015.1  Uncultured bacterium clone A2_92 16S ribosomal RNA ge...  865     0.0
MF114011.1  Uncultured bacterium clone A2_88 16S ribosomal RNA ge...  865     0.0
MF113981.1  Uncultured bacterium clone A2_49 16S ribosomal RNA ge...  865     0.0
MF113975.1  Uncultured bacterium clone A2_42 16S ribosomal RNA ge...  865     0.0
CP014941.1  Rhodococcus sp. BH4, complete genome                      865     0.0
LC133609.2  Rhodococcus sp. JCM 28262 gene for 16S ribosomal RNA,...  865     0.0
LC133620.2  Rhodococcus sp. JCM 28273 gene for 16S ribosomal RNA,...  865     0.0
CP017299.1  Rhodococcus sp. YL-1, complete genome                     865     0.0
```

b)

Figure S4. Sequencing result, plate no 2 a) Blast result of 785F primer; b) Blast result of 907R primer

```
BLASTN 2.6.0+


Reference:
Zheng Zhang, Scott Schwartz, Lukas Wagner, and Webb Miller (2000),
"A greedy algorithm for aligning DNA sequences", J Comput Biol 2000;
7(1-2):203-14.



Database: Nucleotide collection (nt)
           43,634,103 sequences; 150,987,822,336 total letters



Query= 191015-020_L09_P3_785F 1 1453

Length=1453
                                                                      Score     E
Sequences producing significant alignments:                          (Bits)  Value

KY907029.1  Rhodococcus sp. strain N-12 16S ribosomal RNA gene, p...  1280    0.0
KY906993.1  Rhodococcus sp. strain D-46 16S ribosomal RNA gene, p...  1271    0.0
AB288061.1  Rhodococcus boritolerans gene for 16S ribosomal RNA, ...  1269    0.0
AY785731.1  Rhodococcus erythropolis isolate OUCZ20 16S ribosomal...  1269    0.0
KF681164.1  Rhodococcus erythropolis strain 121 16S ribosomal RNA...  1267    0.0
GU565219.1  Rhodococcus sp. BEN 16S ribosomal RNA gene, partial s...  1267    0.0
MF351723.1  Rhodococcus sp. strain YL-1 16S ribosomal RNA gene, p...  1266    0.0
KX156801.1  Rhodococcus sp. 17PEL clone 17PEL 16S ribosomal RNA g...  1266    0.0
CP014941.1  Rhodococcus sp. BH4, complete genome                      1266    0.0
LC133609.2  Rhodococcus sp. JCM 28262 gene for 16S ribosomal RNA,...  1266    0.0
LC133620.2  Rhodococcus sp. JCM 28273 gene for 16S ribosomal RNA,...  1266    0.0
```

a)

```
Query= 191015-020_N09_P3_907R 1 1504

Length=1504
                                                                      Score     E
Sequences producing significant alignments:                          (Bits)  Value

EF459533.1  Nocardiaceae bacterium Ben-13 isolate KFC-53 16S ribo...  1550    0.0
MF351723.1  Rhodococcus sp. strain YL-1 16S ribosomal RNA gene, p...  1548    0.0
CP014941.1  Rhodococcus sp. BH4, complete genome                      1548    0.0
LC133609.2  Rhodococcus sp. JCM 28262 gene for 16S ribosomal RNA,...  1548    0.0
LC133620.2  Rhodococcus sp. JCM 28273 gene for 16S ribosomal RNA,...  1548    0.0
CP017299.1  Rhodococcus sp. YL-1, complete genome                     1548    0.0
LC107443.1  Rhodococcus erythropolis gene for 16S ribosomal RNA, ...  1548    0.0
LC107440.1  Rhodococcus erythropolis gene for 16S ribosomal RNA, ...  1548    0.0
LC133606.1  Rhodococcus sp. JCM 28259 gene for 16S ribosomal RNA,...  1548    0.0
CP012749.1  Rhodococcus sp. 008, complete genome                      1548    0.0
KP412235.1  Uncultured microorganism clone C16 16S ribosomal RNA ...  1548    0.0
KT905696.1  Uncultured bacterium clone Md-107 16S ribosomal RNA g...  1548    0.0
KT905687.1  Uncultured bacterium clone Md-87 16S ribosomal RNA ge...  1548    0.0
KT905659.1  Uncultured bacterium clone Md-46 16S ribosomal RNA ge...  1548    0.0
KT905656.1  Uncultured bacterium clone Md-43 16S ribosomal RNA ge...  1548    0.0
KT905652.1  Uncultured bacterium clone Md-36 16S ribosomal RNA ge...  1548    0.0
KT905639.1  Uncultured bacterium clone Md-9 16S ribosomal RNA gen...  1548    0.0
CP007255.1  Rhodococcus erythropolis R138, complete genome            1548    0.0
```

b)

Figure S5. Sequencing result, agar plate no 3; a) Blast result of 785F primer; b) Blast result of 907R primer

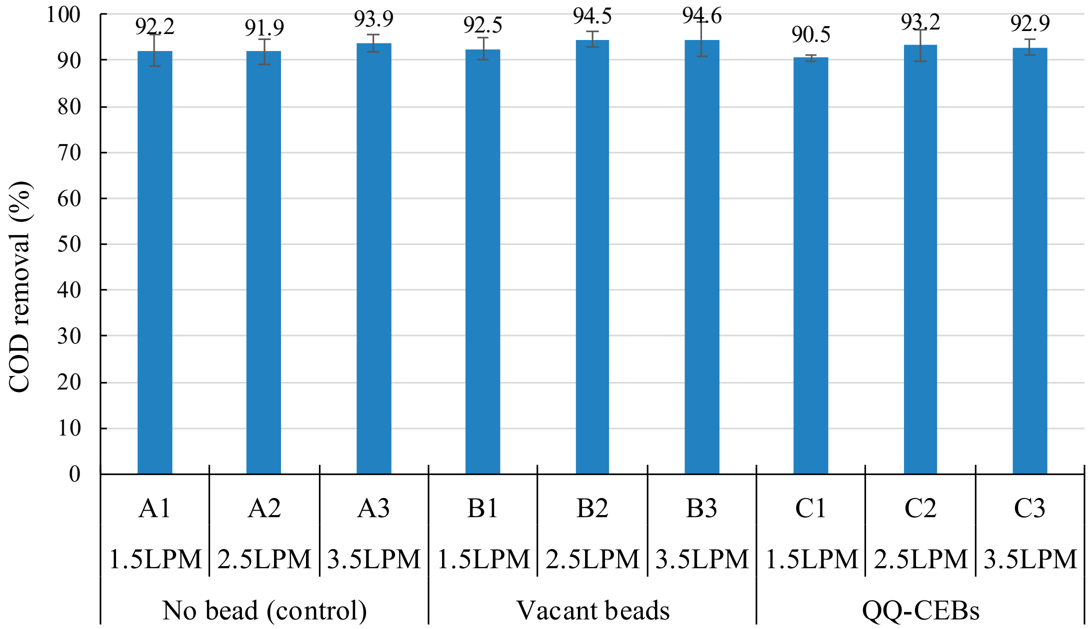

Figure S6. Removal efficiencies for COD

(Error bars : standard error)

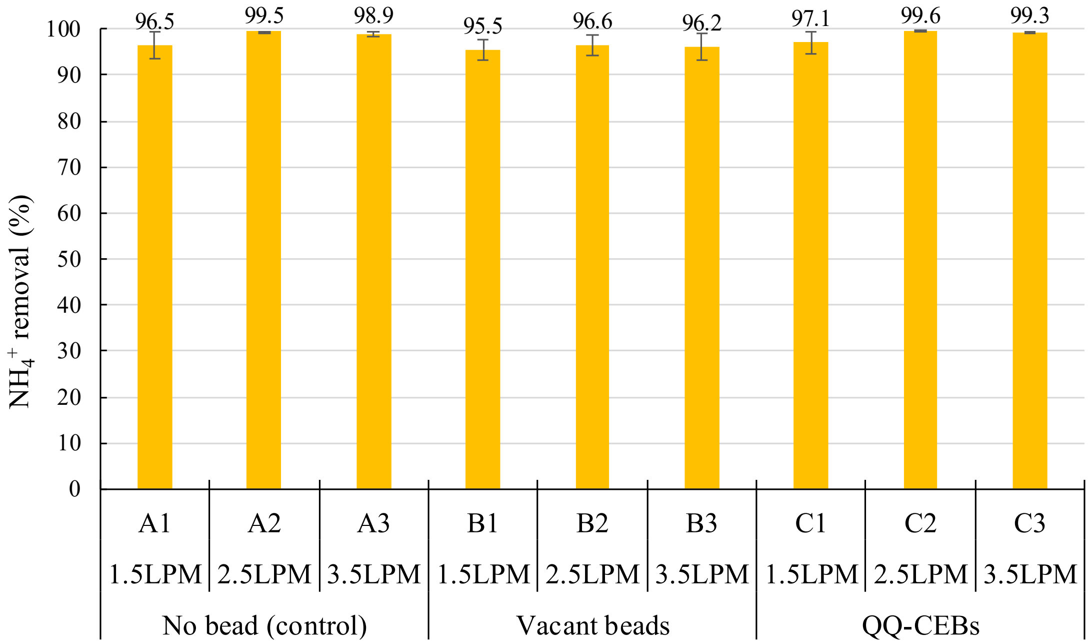

Figure S7. Removal efficiencies for ammonia

(Error bars : standard error)
